# Circular

**Published:** 1856-03

**Authors:** 


*Circular.—American Medical Association.*

The Ninth Annual Meeting of the American Medical Association will be held in the City of Detroit, Michigan, on Tuesday, May 6th, 1856.

The secretaries of all societies and other bodies entitled to representation in the Association, are requested to forward to the undersigned correct lists of their respective delegations, *as soon as they may be appointed;* and it is *earnestly* desired by the Committee of Arrangements, that the appointments be made at as early a period as possible.

The following extracts are from Article 2d of the Constitution:

"Each local society shall have the privilege of sending to the Association one delegate for every ten of its regular resident members, and one for every additional fraction of more than half this number.

"The Faculty of every regularly constituted Medical College or chartered school of medicine, shall have the privilege of sending two delegates. The professional staff of every chartered or municipal hospital, containing a hundred patients or more, shall have the privilege of sending two delegates; and every other permanently organized medical institution, of good standing, shall have the privilege of sending one delegate.

"Delegates, representing the Medical Staff of the United States Army and Navy shall be appointed by the Chiefs of the Army and Navy Medical Bureau. The number of delegates so appointed shall be four from the army medical officers, and an equal number from the navy medical officers."

The latter clause, in relation to delegates from the army and navy, was adopted as an amendment to the Constitution, at the meeting of the Association held in New York, in May, 1853.

Medical Journals, &c., please copy.

WILLIAM BRODIE, M.D., Detroit, Mich.,
*One of the Secretaries.*

